# Inflammation and platelet reactivity during adjunctive colchicine versus aspirin in patients with acute coronary syndrome treated with potent P2Y12 inhibitor

**DOI:** 10.3389/fmed.2024.1349577

**Published:** 2024-04-19

**Authors:** Seung-Yul Lee, Jae Young Cho, Diana A. Gorog, Dominick J. Angiolillo, Kyeong Ho Yun, Jong-Hwa Ahn, Jin-Sin Koh, Yongwhi Park, Seok-Jae Hwang, Jin-Yong Hwang, Jin Won Kim, Yangsoo Jang, Young-Hoon Jeong

**Affiliations:** ^1^Department of Internal Medicine, CHA Bundang Medical Center, Seongnam, Republic of Korea; ^2^Multimodal Imaging and Theranostic Laboratory, Cardiovascular Center, Korea University Guro Hospital, Seoul, Republic of Korea; ^3^Regional Cardiocerebrovascular Center, Wonkwang University Hospital, Iksan, Republic of Korea; ^4^Faculty of Medicine, National Heart and Lung Institute, Imperial College London, London, United Kingdom; ^5^Centre for Health Services Research, School of Life and Medical Sciences, University of Hertfordshire, Hatfield, United Kingdom; ^6^Division of Cardiology, University of Florida College of Medicine, Jacksonville, FL, United States; ^7^Department of Internal Medicine, Gyeongsang National University School of Medicine and Cardiovascular Center, Gyeongsang National University Changwon Hospital, Changwon, Republic of Korea; ^8^Department of Internal Medicine, Gyeongsang National University School of Medicine and Division of Cardiology, Gyeongsang National University Hospital, Jinju, Republic of Korea; ^9^CAU Thrombosis and Biomarker Center, Heart and Brain Hospital, Chung-Ang University Gwangmyeong Hospital and Department of Internal Medicine, Chung-Ang University College of Medicine, Seoul, Republic of Korea

**Keywords:** acute coronary syndrome, percutaneous coronary intervention, colchicine, aspirin, ticagrelor, prasugrel

## Abstract

**Background:**

In patients undergoing percutaneous coronary intervention (PCI), the use of anti-inflammatory therapy with colchicine is associated with a reduction of recurrent ischemic events. The mechanisms of such findings are not fully elucidated.

**Objectives:**

To investigate the effects of colchicine versus aspirin on inflammation and platelet reactivity in patients with acute coronary syndrome (ACS) undergoing PCI.

**Methods:**

This observational study compared laboratory measurements in ACS patients receiving single antiplatelet therapy with ticagrelor or prasugrel plus colchicine (MACT) (*n* = 185) versus conventional dual-antiplatelet therapy (DAPT) with aspirin plus ticagrelor or prasugrel (*n* = 497). The primary outcome was the frequency of high residual inflammation, defined as high-sensitivity C-reactive protein (hs-CRP) ≥2 mg/L at 1 month post-PCI. Multiple sensitivity analyses were performed for the primary outcome, including multivariable adjustment, propensity-score matching, and inverse-probability weighted methods.

**Results:**

One month after PCI, patients treated with MACT had significantly lower levels of hs-CRP compared to those treated with DAPT (0.6 [0.4–1.2] vs. 0.9 [0.6–2.3] mg/L, *p* < 0.001). The frequency of high residual inflammation was also lower in the MACT group (10.8% vs. 27.2%, *p* < 0.001) (odds ratio [95% confidence interval] = 0.33 [0.20–0.54], *p* < 0.001). This effect was consistent across sensitivity analyses. There was no difference in platelet reactivity between MACT and DAPT (49.6 ± 49.0 vs. 51.5 ± 66.4 P2Y_12_ reaction unit [PRU] measured by VerifyNow, *p* = 0.776).

**Conclusion:**

In ACS patients undergoing PCI, MACT was associated with a lower rate of high residual inflammation without increasing platelet reactivity compared to conventional DAPT.

**Clinical trial registration:**

NCT04949516 for MACT pilot trial and NCT04650529 for Gyeongsang National University Hospital registry.

## Introduction

Dual antiplatelet therapy (DAPT) with aspirin and a P2Y_12_ inhibitor has represented the standard-of-care for the treatment and prevention of thrombotic events in patients with an acute coronary syndrome (ACS) (1) undergoing percutaneous coronary intervention (PCI) (2). In this setting, prasugrel or ticagrelor are the preferred oral P2Y_12_ inhibitors, in the absence of contraindications, in light of their superior efficacy over clopidogrel (3, 4). However, DAPT is associated with an increased risk of bleeding which is exacerbated with the prolongation of treatment (5, 6). Given the adverse prognosis associated with bleeding, over the past years a number of novel DAPT regimens have been tested with the goal of mitigating the risk of bleeding while preserving efficacy (7). Among these, P2Y_12_ inhibitor monotherapy after a brief period of DAPT has emerged as a promising bleeding reduction strategy (8–10).

Inflammation plays a crucial role in the pathophysiology of ACS. Persistent elevation of inflammatory markers in ACS patients has been linked to future atherothrombotic events (11–13). Early inhibition of inflammation may be associated with enhanced benefits, particularly in patients with heightened inflammatory levels such as those presenting with ACS (14). Colchicine’s anti-inflammatory effects have been shown to improve cardiovascular outcomes (15–17), it was added to DAPT in ACS patients within the past month or chronic stable patients (18). However, the early effect of colchicine on residual inflammation has not been compared with aspirin, which is recommended after PCI. In addition to its anti-inflammatory effect, colchicine possesses antithrombotic properties which may contributed to its effects on cardiovascular outcomes (19, 20). Recently, the Mono-Antiplatelet and Colchicine Therapy (MACT) pilot trial demonstrated the feasibility of omitting aspiring and maintaining single antiplatelet therapy with ticagrelor or prasugrel combined with colchicine in ACS patients undergoing PCI (21). However, larger studies are warranted to better define the benefits of this approach.

Our study aims to investigate the effects of MACT on inflammation and platelet reactivity in ACS patients, comparing it to conventional DAPT.

## Methods

### Study design and population

**Figure 1:**
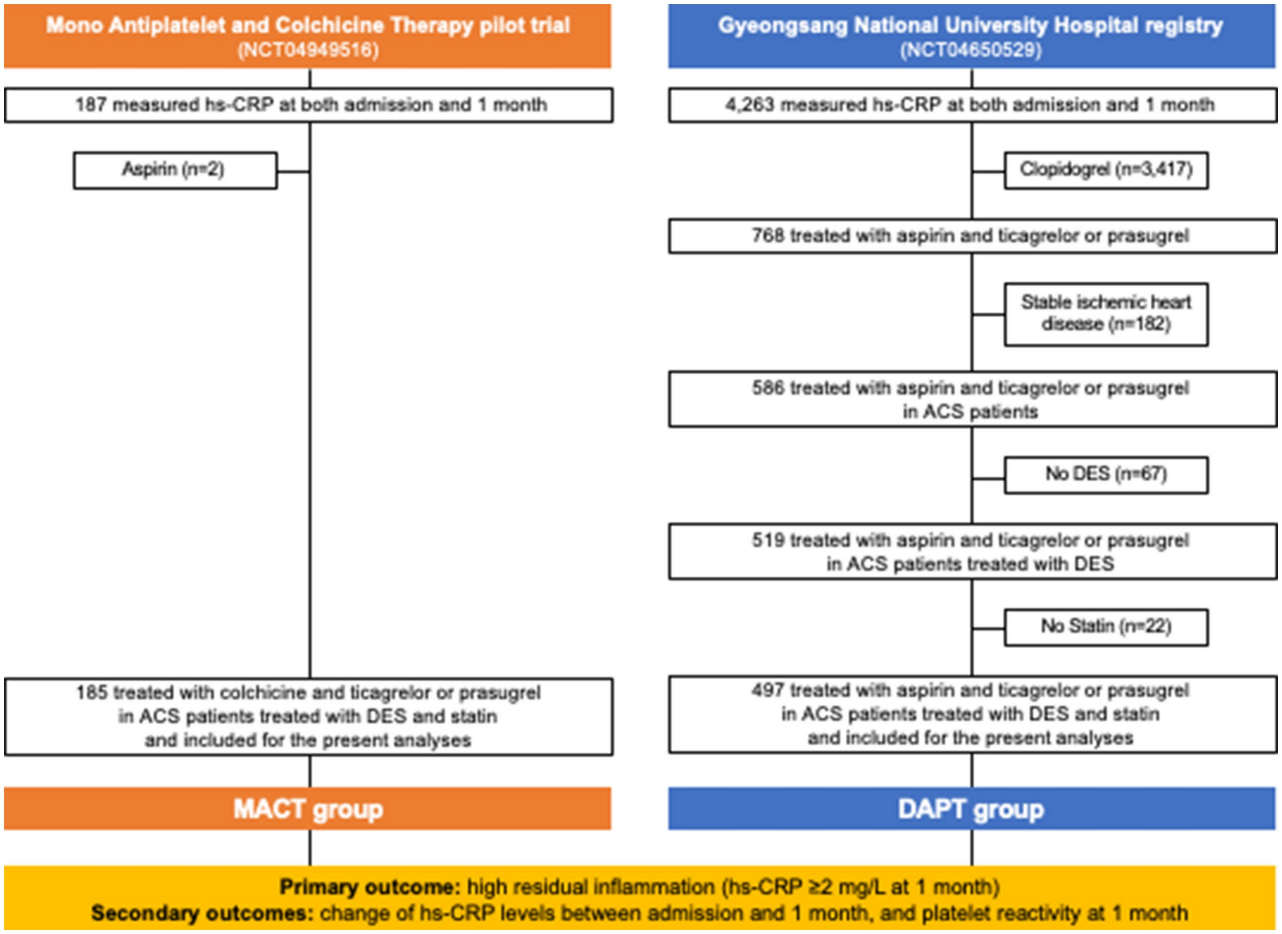
Study flow. ACS, acute coronary syndrome; DAPT, dual antiplatelet therapy; DES, drug-eluting stent; hs-CRP, high-sensitivity C-reactive protein; MACT, mono-antiplatelet and colchicine therapy.

This observational comparative study utilized the data from the MACT pilot trial (NCT04949516) (21) and the Gyeongsang National University Hospital (GNUH) registry (NCT04650529) (22). The MACT pilot trial assessed the feasibility of ticagrelor or prasugrel P2Y_12_ inhibitor monotherapy combined with low-dose colchicine (0.6 mg once daily) in ACS patients immediately after PCI with drug eluting stent (DES) (21). The GNUH registry, a two-center database of PCI-treated patients, examined various hemostatic, vascular, and physiological parameters from January 2010 to November 2018 (22). Figure 1 provides an overview of the study flow. Among a total of 200 patients from the MACT pilot trial, 13 patients did not undergo high-sensitivity C-reactive protein (hs-CRP) examination at 1 month follow-up and 2 patients received aspirin. Thus, 185 ACS patients treated with MACT in the MACT pilot trial, who underwent serial hs-CRP examinations at both admission and 1 month follow-up, were included in the present study (MACT group). From the GNUH registry, 519 ACS patients who underwent DES implantation treated with conventional DAPT, including aspirin (100 mg once daily) plus ticagrelor or prasugrel, were assessed; among them, 22 patients without statin therapy were additionally excluded to minimize bias since all patients from MACT were treated with statins. Therefore, the final analysis included 497 patients (DAPT group). The patients from both groups were not exposed to other anti-inflammatory drugs. Both studies received approval from the institutional review boards, and written informed consent was obtained from all patients in the MACT pilot trial, while consent was waived for the GNUH registry.

### Laboratory measurements

The hs-CRP levels were measured using the UniCel^®^ DxC 800 Synchron^®^ Clinical System (Beckman Coulter, Inc., Brea, CA, United States) with a commercially available enzyme-linked immunosorbent assay. The hs-CRP was measured twice (on admission and 1 month post-PCI). Platelet reactivity at 1 month post-PCI was assessed using the VerifyNow P2Y_12_ assay (Accriva, San Diego, CA, United States). High platelet reactivity (>208 P2Y_12_ reaction units [PRU]) and low platelet reactivity (<85 PRU) were defined based on a consensus document (23). At 1 month, hs-CRP and VeirfyNow P2Y_12_ assessments were performed using blood collected from the antecubital vein at 2 to 6 h after the last drug administration.

### Study outcomes

The primary outcome was the frequency of high residual inflammation, defined as a hs-CRP level of 2 mg or more per liter at 1 month post-PCI (24). Secondary outcomes were the temporal change in inflammation between admission and one month and platelet reactivity at 1 month. In addition, routine laboratory tests, including complete blood count, creatinine, aspartate aminotransferase, alanine aminotransferase, total cholesterol, high-density lipoprotein, and low-density lipoprotein were performed at 1 month. Patients were categorized into the four groups based on the temporal change in hs-CRP levels relative to the threshold of 2 mg/L: (1) persistently high inflammation (hs-CRP ≥ 2 mg/L at both admission and 1 month); (2) aggravated inflammation (hs-CRP < 2 mg/L at admission, but ≥2 mg/L at 1 month); (3) attenuated inflammation (hs-CRP ≥ 2 mg/L at admission, but <2 mg/L at 1 month); and (4) persistently low inflammation (hs-CRP < 2 mg/L at both admission and 1 month) (11).

### Statistical analysis

Continuous variables were presented as either mean ± standard deviation or median (interquartile range) and compared using appropriate statistical tests: Student’s *t*-test, Mann–Whitney U test, or Wilcoxon signed-rank test. Categorical variables were reported as numbers (percentages) and compared using the chi-square test. Logistic regression was employed to calculate the odds ratio (OR) and 95% confidence intervals (Cis) for comparing the primary outcome between the MACT and DAPT groups. The correlation between hs-CRP levels and PRU was determined using Spearman’s rank test.

Multiple sensitivity analyses were performed to adjust for baseline differences, including multivariable regression, propensity-score matching, and inverse-probability weighted analysis. The multivariable model included covariates with *p* < 0.10 in univariate analysis, including dyslipidemia, white blood count, hs-CRP on admission, and colchicine. For propensity-score matching and inverse-probability weighted analysis, variables that could confound the relationship between treatment and outcome were included, such as age, male, body mass index, hypertension, diabetes, dyslipidemia, clinical presentation of acute myocardial infarction, left ventricular ejection fraction <40%, white blood count, hemoglobin, estimated glomerular filtration rate <60 mL/min/1.73 m^2^, total cholesterol, high-density lipoprotein cholesterol, low-density lipoprotein cholesterol, hs-CRP, multi-vessel disease, ticagrelor, and angiotensin blockade. Covariate balance was assessed by calculating the absolute standardized mean differences, which were within ±0.1 across all matched covariates after propensity-score matching or inverse-probability weighted adjustment, suggesting successful balance achievement between the two groups. Procedural characteristics including number of treated vessels, mean stent diameter, or total stent length were additionally included in the regression model after propensity-score matching and inverse-probability weighted adjustment to address residual confounding.

Statistical analyses were performed using SAS (version 9.4; SAS Institute, Cary, NC, United States). All tests were two-sided, and statistical significance was set at *p* < 0.05.

## Results

### Baseline characteristics

Table 1 presents the baseline characteristics of the MACT and DAPT groups. Compared with the DAPT group, the MACT group had a higher proportion of male patients and a higher body mass index, but had lower proportions of dyslipidemia, chronic kidney disease and clinical presentation of acute myocardial infarction. The MACT group also had lower white blood cell counts, but higher hemoglobin levels. Patients in the MACT group showed a lower frequency of multi-vessel disease, and were less likely to receive ticagrelor and angiotensin blockade compared to the DAPT group.

**Table 1:** Baseline characteristics between MACT and DAPT.

	MACT (*n* = 185)	DAPT (*n* = 497)	*p*-value
Age (years)	61.0 ± 10.6	61.4 ± 11.2	0.649
Male	166 (89.7)	392 (78.8)	0.001
Body mass index (kg/m^2^)	25.1 ± 3.1	24.4 ± 3.4	0.012
Risk factors			
Hypertension	95 (51.3)	219 (44.0)	0.089
Diabetes mellitus	57 (30.8)	126 (25.3)	0.152
Dyslipidemia	59 (31.8)	323 (64.9)	<0.001
Chronic kidney disease	27 (14.5)	115 (23.1)	0.014
Current smoking	89 (48.1)	225 (45.2)	0.508
Clinical presentation			<0.001
Unstable angina	53 (28.7)	55 (11.1)	
Acute MI	132 (71.3)	442 (88.9)	
Non–ST-segment elevation MI	50	209	
ST-segment elevation MI	82	233	
Laboratory measurements			
High-sensitivity C-reactive protein (mg/L)	1.3 (0.8–3.7)	1.4 (0.7–3.4)	0.361
High-sensitivity C-reactive protein ≥2 mg/L	76 (41.1)	186 (37.4)	0.383
White blood count (×10^3^/mm^3^)	9.3 ± 3.1	10.0 ± 3.5	0.009
Hemoglobin (g/dL)	14.3 ± 1.6	14.1 ± 1.7	0.048
Creatinine (mg/dL)	0.8 ± 0.2	1.0 ± 1.0	<0.001
Total cholesterol (mg/dL)	184.4 ± 45.5	192.0 ± 45.8	0.053
HDL cholesterol (mg/dL)	43.2 ± 11.8	44.3 ± 13.0	0.317
LDL cholesterol (mg/dL)	113.4 ± 38.7	128.2 ± 42.0	<0.001
LV ejection fraction <40%	12 (6.5)	25 (5.0)	0.455
Procedural characteristics			
Multi-vessel disease	54 (29.1)	223 (44.8)	<0.001
Use of drug-eluting stent	185 (100.0)	497 (100.0)	1.000
Number of treated vessels	1.2 ± 0.4	1.2 ± 0.4	0.764
Number of implanted stents	1.4 ± 0.7	1.4 ± 0.7	0.534
Mean stent diameter (mm)	3.1 ± 0.3	3.2 ± 0.4	0.013
Total stent length (mm)	36.8 ± 21.5	36.4 ± 21.4	0.841
Discharge medications			
Aspirin, 100 mg daily	0 (0)	497 (100.0)	<0.001
Colchicine, 0.6 mg daily	185 (100.0)	0 (0)	
High-intensity statin	143 (77.3)	381 (76.7)	0.861
Type of P2Y_12_ inhibitor			<0.001
Ticagrelor	94 (50.8)	390 (78.4)	
Prasugrel	91 (49.2)	107 (21.6)	
Angiotensin blockade	127 (68.6)	413 (83.1)	<0.001
Beta blocker	129 (69.7)	381 (76.6)	0.063

High-intensity statin indicated 40 mg or more of atorvastatin or 20 mg or more of rosuvastatin. DAPT, dual antiplatelet therapy; HDL, high-density lipoprotein; LDL, low-density lipoprotein; LV, left ventricle; MACT, mono antiplatelet and colchicine therapy; MI, myocardial infarction.

### Residual inflammation

**Figure 2:**
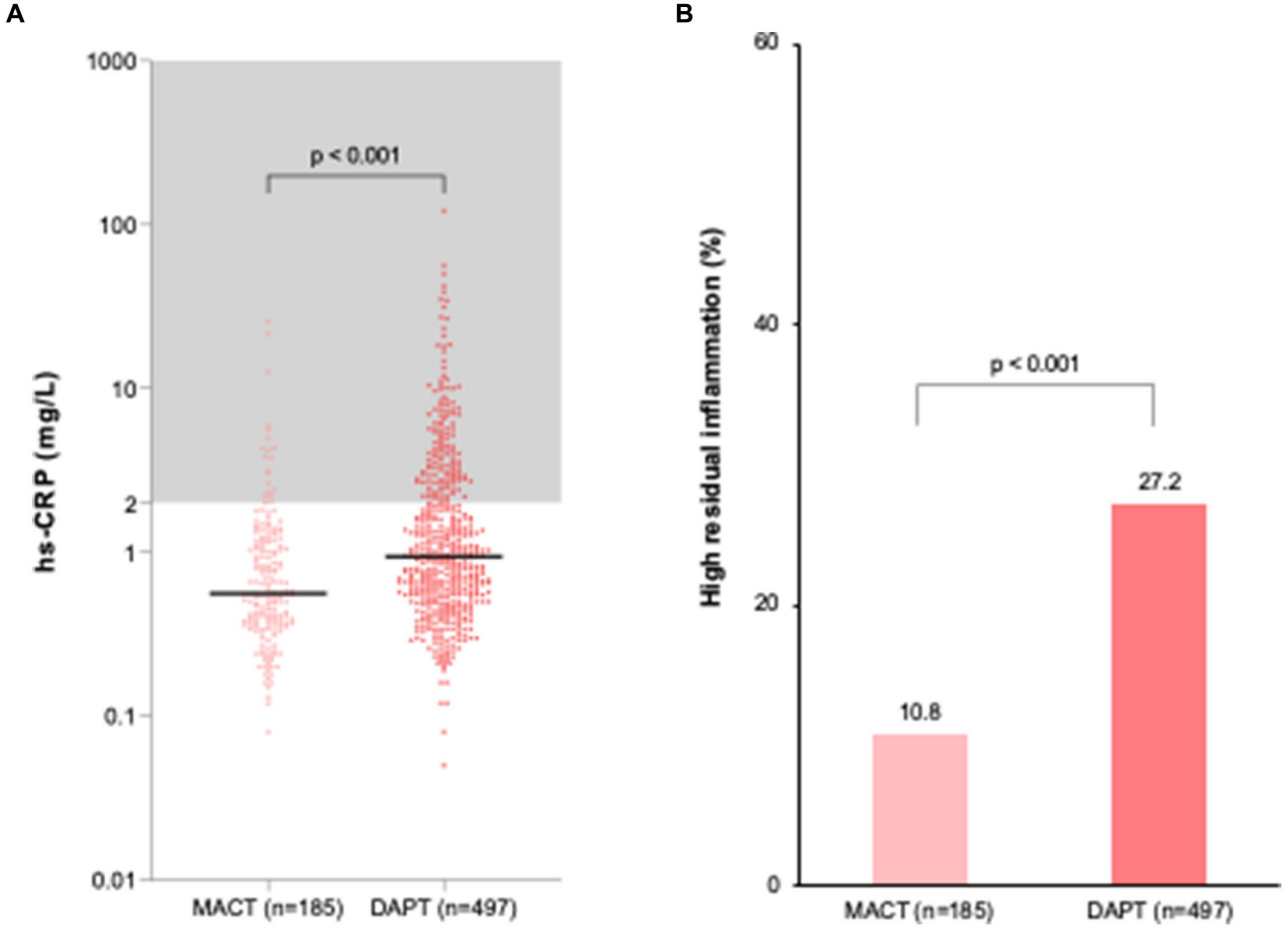
Residual inflammation at 1 month after PCI in ACS patients. **(A)** hs-CRP levels; **(B)** Frequency of high residual inflammation. DAPT, dual antiplatelet therapy; hs-CRP, high-sensitivity C-reactive protein; MACT, mono-antiplatelet and colchicine therapy.

Figure 2 displays residual inflammation at 1 month post-PCI. The MACT group had lower hs-CRP levels (0.6 [0.4–1.2] vs. 0.9 [0.6–2.3] mg/L, *p* < 0.001) (Figure 2A) and a lower frequency of high residual inflammation (10.8% vs. 27.2%, *p* < 0.001, primary outcome) (Figure 2B) compared to the DAPT group. In a multivariable regression analysis including variables with *p* < 0.1 from univariate regression analysis (Supplementary Table S1), the MACT vs. DAPT strategy was an independent determinant of high residual inflammation (hs-CRP ≥ 2 mg/L at 1 month) (odds ratio [95% confidence interval] = 0.35 [0.21–0.59], *p* < 0.001) (Supplementary Table S2).

Table 2 presents the risk of high residual inflammation during MACT vs. DAPT from multiple sensitivity analyses, which consistently showed its lower risk. Multiple sensitivity analyses included multivariable regression analysis (Supplementary Table S2), propensity-score matching analysis (Supplementary Tables S3, S4), and inverse-probability weighted analysis (Supplementary Table S4).

**Table 2:** High residual inflammatory risk of MACT from multiple sensitivity analyses.

	Odds ratio (95% confidence interval)	*p*-value
Univariate	0.33 (0.20–0.54)	<0.001
Multivariable	0.35 (0.21–0.59)	<0.001
Propensity-score matching	0.36 (0.19–0.70)	0.002
Inverse-probability weighted	0.23 (0.17–0.32)	<0.001

### Temporal change in inflammation

**Figure 3:**
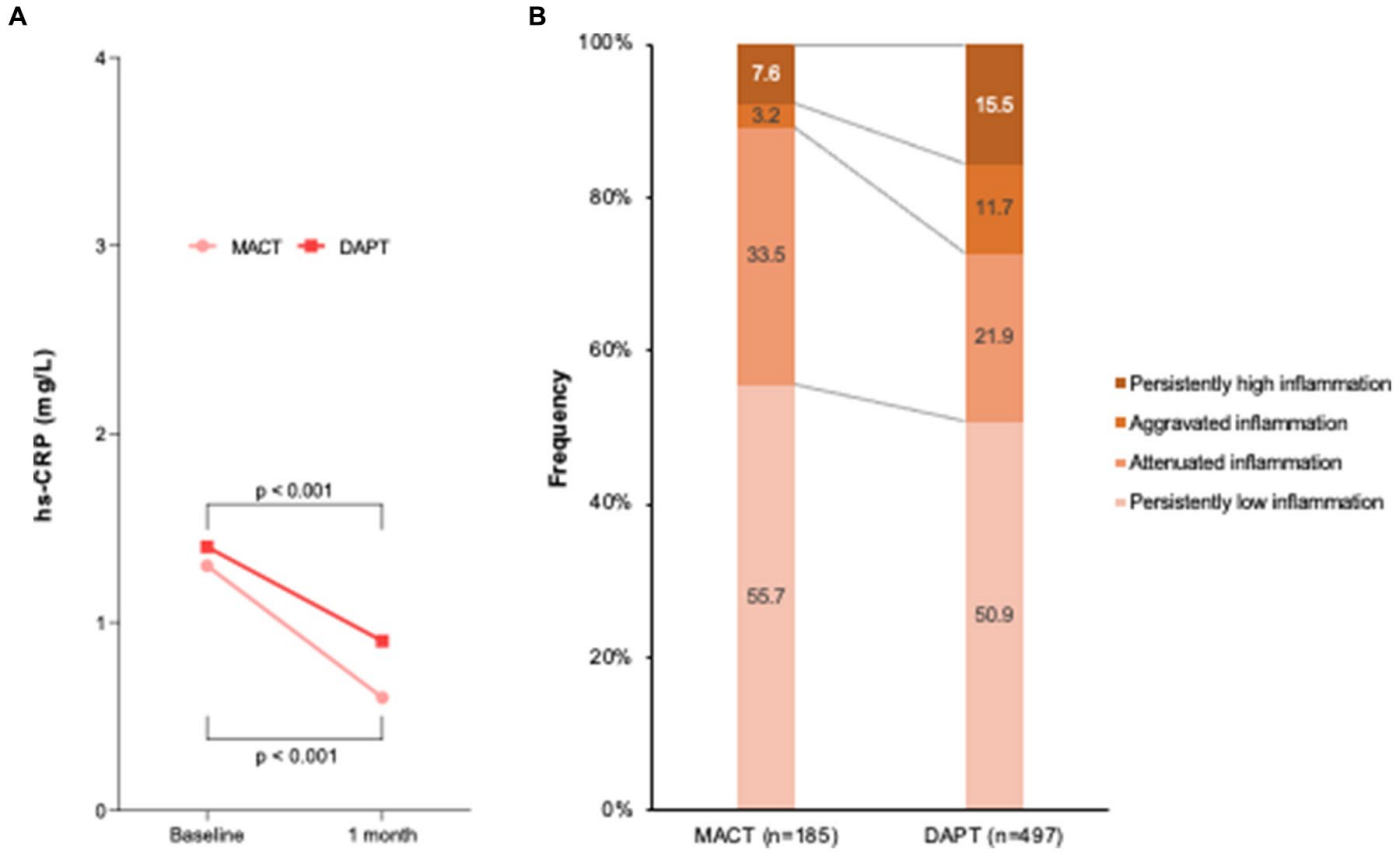
Temporal change in inflammation between admission and 1 month follow-up. **(A)** hs-CRP levels; **(B)** Frequency of temporal inflammation criteria. DAPT, dual antiplatelet therapy; hs-CRP, high-sensitivity C-reactive protein; MACT, mono-antiplatelet and colchicine therapy.

Figure 3 illustrates the temporal change in inflammation between admission and 1 month follow-up. At admission, hs-CRP levels were not different between the MACT and DAPT groups (1.3 [0.8–3.7] vs. 1.4 [0.7–3.4] mg/L, *p* = 0.361). The significant decrease in hs-CRP levels was shown in both groups during one month (Figure 3A). However, the difference in reduction in hs-CRP levels was greater in the MACT vs. DAPT group (△ − 0.6 [−2.5–−0.1] vs. △ − 0.1 [−1.7–0.3] mg/L, *p* < 0.001), which was accompanied by the lower rate of persistently high inflammation (7.6% vs. 15.5%, *p* = 0.007) (Figure 3B).

### Subgroup analysis

**Figure 4:**
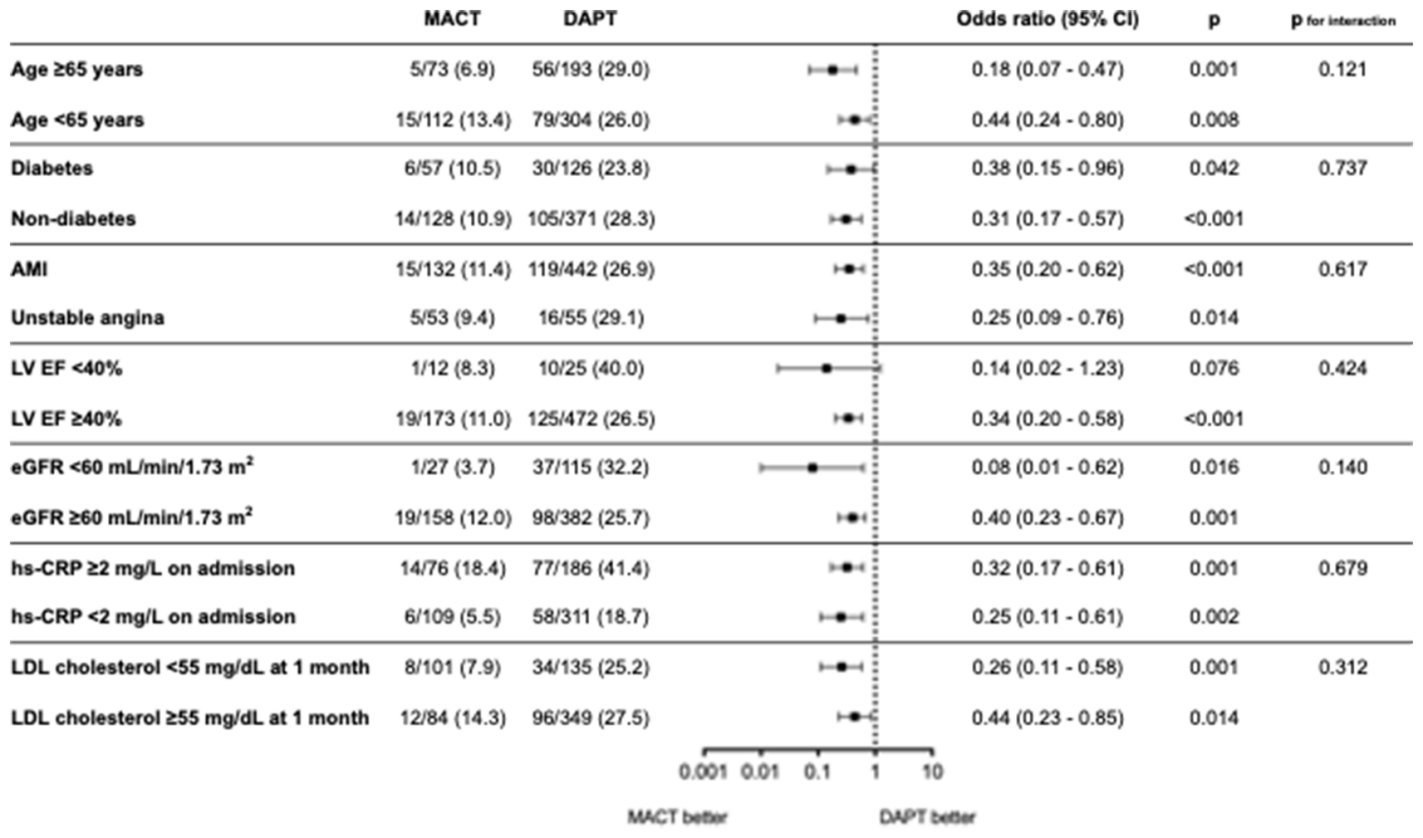
Risk of high residual inflammation between MACT and DAPT across the subgroups. AMI, acute myocardial infarction; CI, confidence interval; DAPT, dual antiplatelet therapy; EF, ejection fraction; eGFR, estimated glomerular filtration rate; hs-CRP, high-sensitivity C-reactive protein; LDL, low-density lipoprotein; LV, left ventricle; MACT, mono-antiplatelet and colchicine therapy.

Figure 4 shows a forest plot indicating the risk of high residual inflammation between the MACT and DAPT groups according to subgroups. The lower risk of high residual inflammation observed in the MACT group remained consistent across different baseline characteristics and low-density lipoprotein-cholesterol level at 1 month, without any significant interaction.

### Platelet reactivity

**Figure 5:**
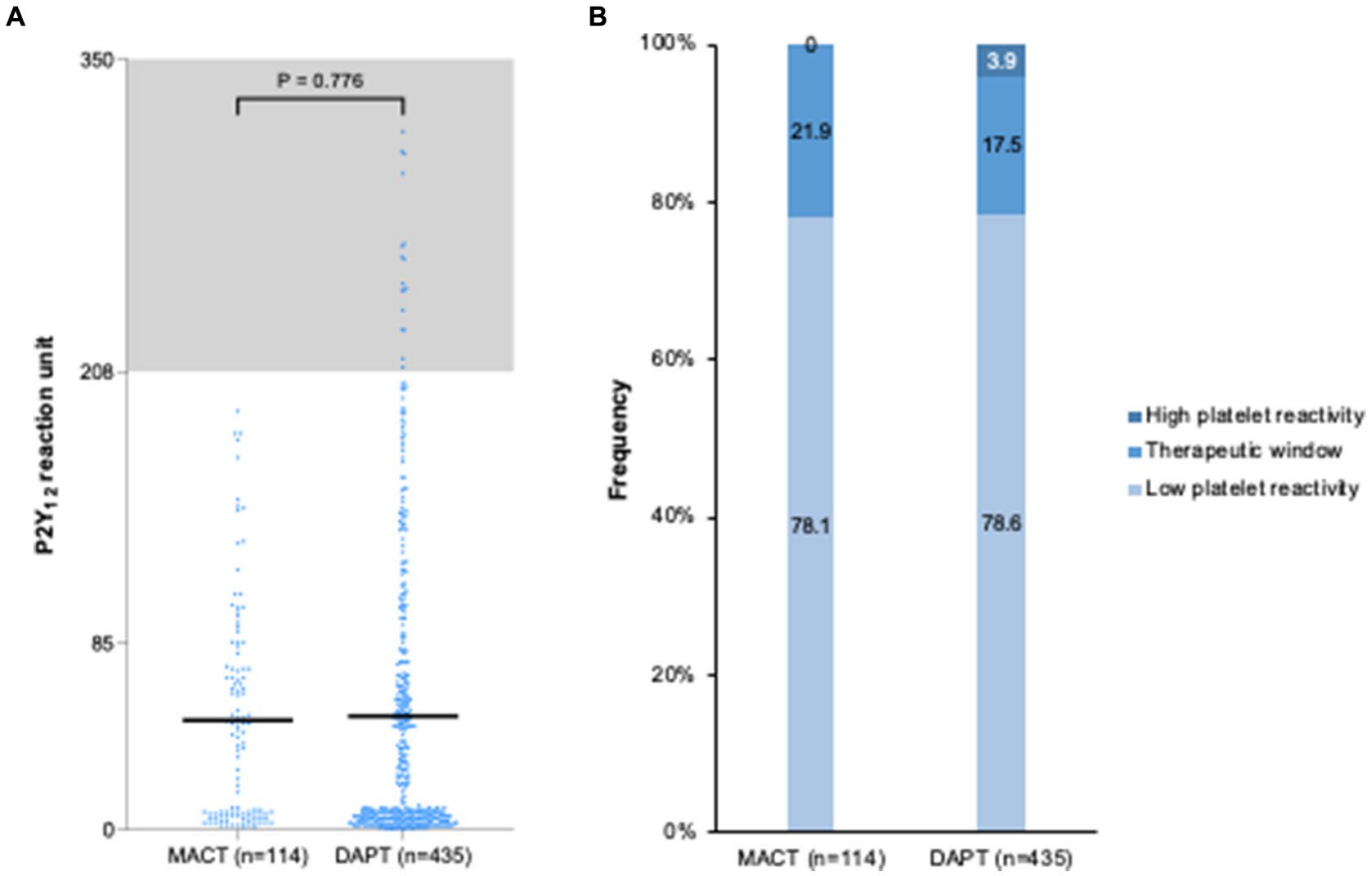
Platelet reactivity at one month after PCI in ACS patients. **(A)** P2Y_12_ reaction unit levels; **(B)** Frequency of platelet reactivity. DAPT, dual antiplatelet therapy; MACT, mono-antiplatelet and colchicine therapy.

Figure 5 presents platelet reactivity at 1 month in the MACT (*n* = 114) and DAPT (*n* = 435) groups. The PRU levels were similar between groups (49.6 ± 49.0 vs. 51.5 ± 66.4 PRU, *p* = 0.776) (Figure 5A). The frequency of platelet reactivity criteria was not significantly different (*p* = 0.066), without high platelet reactivity in the MACT group (Figure 5B). Neither the hs-CRP levels at admission (*r* = 0.029, *p* = 0.492) nor at the 1 month follow-up (*r* = −0.011, *p* = 0.791) were correlated with PRU values.

## Discussion

This analysis represents the first comparison between colchicine and aspirin, along with a potent P2Y_12_ inhibitor on their early effects on residual inflammation and platelet reactivity in ACS patients undergoing PCI. The key findings of this analysis are as follows: (1) colchicine reduced the risk of high residual inflammation (hs-CRP ≥ 2 mg/L) in comparison to aspirin; (2) the frequency of persistently high inflammation (hs-CRP ≥ 2 mg/L at both admission and 1 month) was also lower in colchicine therapy; (3) the lower risk of high residual inflammation with colchicine was consistent across subgroups such as age, diabetes, clinical presentation, systolic heart function, renal insufficiency, baseline inflammatory activity, and residual low-density lipoprotein cholesterol level; and (4) the addition of colchicine to a potent P2Y_12_ inhibitor achieved a similar antiplatelet effect to standard DAPT, as assessed by ADP-induced platelet reactivity using the VerifyNow PRU assay. These findings suggest that colchicine may offer advantages over aspirin in terms of reducing residual inflammation without compromising the antiplatelet effects observed with standard DAPT in ACS patients undergoing PCI.

The rationale for the MACT strategy is based on two key aspects. First, in presence of potent P2Y_12_ inhibition with standard doses of prasugrel or ticagrelor, aspirin has shown to have limited adjunctive antithrombotic efficacy as shown in both pharmacodynamic and clinical studies; moreover, omitting aspirin markedly reduces bleeding (25–29). Second, the anti-inflammatory effects of colchicine which may be of enhanced benefit early after an ACS presentation when inflammation is heightened (14). By combining these concepts, MACT aims to reduce both ischemic and bleeding events compared to conventional DAPT. Colchicine has emerged as a potential candidate with more specific anti-inflammatory efficacy than aspirin. Its anti-inflammatory effects translate into improved ischemic outcomes (15, 16). Although a previous MACT pilot trial demonstrated the feasibility of this strategy in ACS patients undergoing PCI, it did not compare the effects of MACT on residual inflammation and platelet reactivity, both of which are established predictors for future atherothrombotic events, with conventional DAPT (21). The present analysis reveals a significant decrease in high residual inflammation among patients treated with MACT, regardless of baseline inflammatory activity. Specifically, the statistical significance of baseline hs-CRP level was attenuated after adjusting colchicine therapy in the multivariable model. This finding is particularly important as persistently high inflammation has been associated with higher rates of mortality and myocardial infarction (11, 12, 30). Furthermore, the efficacy of colchicine therapy appears to be consistent across subgroups and unaffected by clinical factors. However, there remains controversy regarding the clinical benefit of low-dose colchicine according to hs-CRP levels in patients with acute myocardial infarction. Two randomized trials reported inconsistent results despite a meta-analysis suggesting a reduction in hs-CRP and interleukin-6 levels in patients with coronary artery disease (17, 31, 32).

Colchicine disrupts the cellular cytoskeleton by inhibiting mitosis and intracellular transport through the suppression of tubulin polymerization. This can explain why colchicine can inhibit platelet aggregation, degranulation, and the formation of platelet-derived extracellular vesicles. Colchicine also inhibits leucocyte recruitment and activity in inflamed vascular areas (33, 34). Additionally, colchicine hinders the activation of the nucleotide-binding oligomerization domain, leucine-rich repeat, and pyrin domain-containing protein 3 inflammasome, as well as the secretion of tumor necrosis factor-α (33, 34). Although there is lack of data regarding plaque stabilization with colchicine therapy, an observational study conducted by Vaidya et al. found that low-dose colchicine therapy led to beneficial modifications in coronary plaque, manifested by a decrease in both low attenuation plaque volume and hs-CRP levels when compared to controls (35). In addition, colchicine has been shown to modulate low-grade inflammation of adipose tissue (34) and may also modify the inflammatory activity of epicardial adipose tissue, considering its pro-inflammatory effects and contribution to high-risk plaque progression (36). However, further research is necessary to comprehensively investigate the action of anti-inflammatory therapy for the treatment of coronary artery disease and explore the potential role of colchicine in current DAPT for patients undergoing PCI.

The present study found no difference in ADP-mediated platelet reactivity, assessed by PRU at 1 month post-PCI, between colchicine and aspirin when combined with a potent P2Y_12_ inhibitor. Colchicine has been reported to directly inhibit the release of platelet-derived microparticles and the expression of platelet activation markers (37, 38). Additionally, reduction of residual inflammation by colchicine therapy may impact platelet reactivity, as elevated levels of inflammatory markers like hs-CRP and fibrinogen have been linked to increased platelet reactivity (39, 40). Although the present study did not demonstrate a correlation between hs-CRP and PRU measures, a previous study demonstrated that higher hs-CRP levels were associated with greater platelet reactivity in PCI patients treated with clopidogrel (41). Therefore, the current findings suggest that ticagrelor or prasugrel might be less susceptible to the pro-aggregatory effects of inflammation compared to clopidogrel.

### Limitations

The present analysis was performed using two cohorts with different characteristics. Confounding factors or unmeasured variables such as the presence of peripheral artery disease may have affected the present results despite multiple sensitivity analyses. Additionally, potential selection bias may have led to biased results due to the different nature of the studies being compared. The baseline PRU levels were not adjusted because they were not measured in the MACT trial. However, most patients included in this study were naïve to P2Y_12_ inhibitors on admission. The MACT cohort was also smaller than the DAPT cohort. Only one platelet function assay using ADP agonist was used. Therefore, although the present study showed that colchicine has a limited effect on ADP-induced platelet reactivity, it cannot be excluded that other platelet signaling pathways may be affected, thus warranting more comprehensive studies to assess the effects of colchicine on platelet biology.

## Conclusion

Compared to conventional DAPT, MACT is associated with a lower risk of high residual inflammation and similar ADP-induced platelet reactivity at 1 month after PCI in ACS patients. Larger studies are warranted to understand the clinical implications of these findings.

## Data availability statement

The data underlying this article will be shared on reasonable request to the corresponding author.

## Ethics statement

The studies involving humans were approved by Wonkwang University Institutional Review Board. The studies were conducted in accordance with the local legislation and institutional requirements. The participants provided their written informed consent to participate in this study.

## Author contributions

S-YL: Conceptualization, Data curation, Formal analysis, Investigation, Methodology, Resources, Writing – original draft. JC: Formal analysis, Investigation, Methodology, Resources, Writing – original draft. DG: Writing – review & editing. DA: Writing – review & editing. KY: Writing – review & editing. J-HA: Writing – review & editing. J-SK: Writing – review & editing. YP: Writing – review & editing. S-JH: Writing – review & editing. J-YH: Writing – review & editing. JK: Writing – review & editing. YJ: Writing – review & editing. Y-HJ: Data curation, Funding acquisition, Investigation, Methodology, Resources, Supervision, Writing – review & editing.
