# Supplementary material for: Inflammation and platelet reactivity during adjunctive colchicine versus aspirin in patients with acute coronary syndrome treated with potent P2Y12 inhibitor
Source: Front Med (Lausanne). 2024 Apr 19;11:1349577. doi: 10.3389/fmed.2024.1349577 (PMC11151890; doi:10.3389/fmed.2024.1349577)
Supplement: Supplementary file 1 [file Table_1.docx]

**SUPPLEMENTAL MATERIAL**

**Inflammation and platelet reactivity during adjunctive colchicine versus aspirin in patients with acute coronary syndrome treated with potent P2Y12 inhibitor**

This appendix has been provided by the authors to give readers additional information about their work.

**Supplemental Tables**

Table S1. Univariate regression analysis for the predictors of high residual inflammation (hs-CRP ≥ 2 mg/L at 1-month follow-up)

Table S2. Predictors for high residual inflammation from multivariable regression analysis

Table S3. Baseline characteristics between MACT and DAPT in propensity-score matched population

Table S4. Standardized differences of variables used in propensity-score matched and inverse-probability weighted analyses

**Supplemental Table 1. Univariate regression analysis for the predictors of high residual inflammation (hs-CRP ≥ 2 mg/L at 1-month follow-up)**

|  | **Odds ratio (95% confidence interval)** | ***p*** |
| --- | --- | --- |
| Age (years) | 1.01 (0.99 – 1.02) | 0.235 |
| Male | 1.07 (0.67 – 1.71) | 0.779 |
| Body mass index (kg/m^2^) | 0.96 (0.91 – 1.01) | 0.131 |
| Hypertension | 0.81 (0.56 – 1.16) | 0.243 |
| Diabetes mellitus | 0.78 (0.52 – 1.19) | 0.249 |
| Dyslipidemia | 1.37 (0.95 – 1.98) | 0.091 |
| Current smoking | 1.17 (0.82 – 1.67) | 0.395 |
| Chronic kidney disease | 1.32 (0.86 – 2.02) | 0.198 |
| Acute MI | 1.26 (0.75 – 2.11) | 0.375 |
| LV ejection fraction < 40% | 1.47 (0.71 – 3.05) | 0.298 |
| White blood cell count (×10^3^/mm^3^) | 1.07 (1.01 – 1.12) | 0.012 |
| Hemoglobin (g/dL) | 0.95 (0.85 – 1.05) | 0.282 |
| Total cholesterol (mg/dL) | 1.01 (1.00 – 1.01) | 0.018 |
| HDL cholesterol (mg/dL) | 1.00 (0.98 – 1.01) | 0.735 |
| LDL cholesterol (mg/dL) | 1.01 (1.00 – 1.01) | 0.008 |
| hs-CRP, baseline (mg/L) | 1.01 (1.00 – 1.01) | 0.076 |
| Multi-vessel disease | 1.32 (0.92 – 1.89) | 0.135 |
| Number of treated vessels | 1.03 (0.7 – 1.52) | 0.870 |
| Number of implanted stents | 1.04 (0.82 – 1.33) | 0.733 |
| Mean stent diameter (mm) | 0.95 (0.64 – 1.43) | 0.813 |
| Total stent length (mm) | 1.00 (0.99 – 1.01) | 0.802 |
| Medications |  |  |
| Colchicine vs. aspirin | 0.33 (0.20 – 0.54) | <0.001 |
| High-intensity statin vs. others | 0.87 (0.57 – 1.32) | 0.503 |
| Ticagrelor vs. prasugrel | 1.13 (0.76 – 1.69) | 0.546 |
| Angiotensin blockade | 1.12 (0.72 – 1.76) | 0.609 |
| Beta blocker | 1.10 (0.72 – 1.67) | 0.660 |

High-intensity statin indicated 40 mg or more of atorvastatin or 20 mg or more of rosuvastatin.

HDL, high-density lipoprotein; hs-CRP, high sensitivity C-reactive protein; LDL, low-density lipoprotein; LV, left ventricle; MI, myocardial infarction.

**Supplemental Table 2. Predictors for high residual inflammation from multivariable regression analysis**

|  | **Odds ratio (95% CI)** | ***p*** |
| --- | --- | --- |
| Dyslipidemia | 1.12 (0.76 – 1.65) | 0.576 |
| White blood cell count, baseline (per 10^3^/mm^3^ increase) | 1.05 (1.00 – 1.11) | 0.050 |
| hs-CRP, baseline (per 1-mg/L increase) | 1.01 (1.00 – 1.01) | 0.107 |
| Colchicine vs. aspirin | 0.35 (0.21 – 0.59) | <0.001 |

CI, confidence interval; hs-CRP, high sensitivity C-reactive protein.

**Supplemental Table 3. Baseline characteristics between MACT and DAPT in propensity-score matched population**

|  | **MACT group**  **(n=138)** | **DAPT group**  **(n=138)** | ***p*** |
| --- | --- | --- | --- |
| Age (years) | 62.4 ± 10.7 | 63.5 ± 11.0 | 0.410 |
| Male | 119 (86.2) | 115 (83.3) | 0.502 |
| Body mass index (kg/m^2^) | 24.9 ± 3.2 | 24.7 ± 3.5 | 0.692 |
| **Risk factors** |  |  |  |
| Hypertension | 72 (52.1) | 78 (56.5) | 0.468 |
| Diabetes mellitus | 40 (28.9) | 37 (26.8) | 0.687 |
| Dyslipidemia | 53 (38.4) | 52 (37.6) | 0.901 |
| Chronic kidney disease | 24 (17.3) | 28 (20.2) | 0.538 |
| Current smoking | 59 (42.7) | 60 (43.4) | 0.903 |
| **Clinical presentation** |  |  | 0.667 |
| Unstable angina | 30 (21.7) | 33 (23.9) |  |
| Acute MI | 108 (78.3) | 105 (76.1) |  |
| Non–ST-segment elevation MI | 40 | 43 |  |
| ST-segment elevation MI | 68 | 62 |  |
| **Laboratory measurements** |  |  |  |
| High-sensitivity C-reactive protein (mg/L) | 1.3 (0.7 – 4.2) | 1.2 (0.7 – 3.4) | 0.353 |
| White blood cell count (×10^3^/mm^3^) | 9.5 ± 3.3 | 9.3 ± 3.3 | 0.721 |
| Hemoglobin (g/dL) | 14.2 ± 1.6 | 14.0 ± 1.6 | 0.406 |
| Creatinine (mg/dL) | 0.8 ± 0.2 | 0.9 ± 0.4 | 0.080 |
| Total cholesterol (mg/dL) | 182.2 ± 46.8 | 177.7 ± 41.1 | 0.405 |
| HDL cholesterol (mg/dL) | 43.7 ± 11.6 | 43.7 ± 12.8 | 0.996 |
| LDL cholesterol (mg/dL) | 111.7 ± 39.1 | 111.5 ± 31.8 | 0.959 |
| LV ejection fraction <40% | 12 (8.7) | 9 (6.52) | 0.495 |
| **Procedural characteristics** |  |  |  |
| Multi-vessel disease | 50 (36.2) | 47 (34.0) | 0.705 |
| Use of drug-eluting stent | 138 (100.0) | 138 (100.0) | 1.000 |
| Number of treated vessels | 1.2 ± 0.5 | 1.1 ± 0.4 | 0.260 |
| Number of implanted stents | 1.4 ± 0.7 | 1.3 ± 0.6 | 0.412 |
| Stent diameter (mm) | 3.1 ± 0.3 | 3.2 ± 0.4 | 0.007 |
| Total stent length (mm) | 39.1 ± 23.6 | 34.6 ± 17.6 | 0.073 |
| **Discharge medications** |  |  |  |
| Statin | 138 (100.0) | 138 (100.0) | 1.000 |
| Type of P2Y_12_ inhibitor |  |  | 0.805 |
| Ticagrelor | 83 (60.1) | 85 (61.6) |  |
| Prasugrel | 55 (39.9) | 53 (38.4) |  |
| Angiotensin blockade | 98 (71.0) | 98 (71.0) | 1.000 |
| Beta blocker | 98 (71.0) | 97 (70.2) | 0.894 |

DAPT, dual antiplatelet therapy; HDL, high-density lipoprotein; LDL, low-density lipoprotein; LV, left ventricle; MACT, mono-antiplatelet and colchicine therapy; MI, myocardial infarction.

**Supplemental Table 4. Standardized differences of variables used in propensity-score matching and inverse-probability weighted analyses**

|  | **Standardized differences** | | |
| --- | --- | --- | --- |
|  | **Unadjusted** | **Propensity-score matching** | **inverse-probability weighted** |
| Age (years) | -0.024 | -0.099 | -0.015 |
| Male | -0.278 | -0.081 | -0.006 |
| Body mass index (kg/m^2^) | 0.214 | 0.049 | -0.002 |
| Hypertension | -0.154 | 0.087 | 0.010 |
| Diabetes mellitus | -0.140 | -0.048 | 0.071 |
| Dyslipidemia | 0.713 | -0.015 | 0.007 |
| Acute MI | 0.453 | -0.055 | 0.042 |
| LV ejection fraction <40% | -0.073 | -0.094 | 0.096 |
| White blood cell count (×10^3^/mm^3^) | -0.233 | 0.043 | 0.011 |
| Hemoglobin (g/dL) | 0.156 | 0.099 | 0.088 |
| Creatinine clearance <60 mL/min | 0.218 | 0.074 | 0.078 |
| Total cholesterol (mg/dL) | -0.184 | 0.096 | 0.098 |
| HDL cholesterol (mg/dL) | -0.088 | 0.001 | 0.043 |
| LDL cholesterol (mg/dL) | -0.383 | 0.005 | 0.049 |
| High-sensitivity C-reactive protein (mg/L) | -0.039 | 0.048 | -0.031 |
| Multi-vessel disease | 0.325 | -0.045 | -0.081 |
| Ticagrelor vs. prasugrel | 0.612 | 0.031 | -0.048 |
| Angiotensin blockade | 0.349 | 0.000 | 0.061 |

HDL, high-density lipoprotein; LDL, low-density lipoprotein; LV, left ventricle; MI, myocardial infarction.
